# Active Surveillance for Papillary Thyroid Microcarcinoma: Challenges and Prospects

**DOI:** 10.3389/fendo.2018.00736

**Published:** 2018-12-14

**Authors:** Shuai Xue, Peisong Wang, Zachary A. Hurst, Yi Seok Chang, Guang Chen

**Affiliations:** ^1^Thyroid Surgery Department, The First Hospital of Jilin University, Changchun, China; ^2^Department of Physiology and Cell Biology, The Ohio State University, Columbus, OH, United States

**Keywords:** active surveillance, papillary thyroid microcarcinoma, imaging, biomarker, recurrence

## Abstract

Active surveillance (AS) can be considered as an alternative to immediate surgery in low-risk papillary thyroid microcarcinoma (PTMC) without clinically apparent lymph nodes, gross extrathyroidal extension (ETE), and/or distant metastasis according to American Thyroid Association. However, in the past AS has been controversial, as evidence supporting AS in the management of PTMC was scarce. The most prominent of these controversies included, the limited accuracy and utility of ultrasound (US) in the detection of ETE, malignant lymph node involvement or the advent of novel lymph node malignancy during AS, and disease progression. We summarized publications and indicated: (1) US, performer-dependent, could not accurately diagnose gross ETE or malignant lymph node involvement in PTMC. However, the combination of computed tomography and US provided more accurate diagnostic performance, especially in terms of selection sensitivity. (2) Compared to immediate surgery patients, low-risk PTMC patients had a slightly higher rate of lymph node metastases (LNM), although the overall rate for both groups remained low. (3) Recent advances in the sensitivity and specificity of imaging and incorporation of diagnostic biomarkers have significantly improved confidence in the ability to differentiate indolent vs. aggressive PTMCs. Our paper reviewed current imagings and biomarkers with initial promise to help select AS candidates more safely and effectively. These challenges and prospects are important areas for future research to promote AS in PTMC.

## Introduction

In an early era of medicine, cancer was diagnosed at advanced and incurable stages due to poor diagnostic technologies and limited therapeutic options. High mortality from cancer evoked fear and promoted “early detection and curative treatment” as the holy grail for oncologists (1). Improved technology shifted cancer diagnosis to earlier time-points at less advanced stages, the so called “stage migration.” Consequently, detection of sub-clinical small cancers became feasible (1, 2). Attributable to improvements in early detection and subsequent increased the number of novel diagnoses, the incidence of localized, *in situ*, cancers (particularly thyroid, melanoma, and kidney) doubled or tripled between 1975 and 2005 according to SEER database (https://seer.cancer.gov/). Despite the increased incidence, thyroid cancer mortality remains stable (3). Moreover, owing to indolent behavior and favorable prognosis of these cancers, high frequency of occult microcarcinoma in autopsy studies has been also reported (4–9). These evidences indicated that doctors were diagnosing and treating many inert cancers, which would never cause any harm or threaten patient's lives even if left untreated.

Concerns about overdiagnosis and overtreatment lead to the introduction of active surveillance (AS) for indolent cancers, such as low-risk prostate cancer and papillary thyroid cancer, whose 5-year survival rates approached 100%(10). AS has become a routine treatment strategy for localized prostate cancer (11–13). A randomized controlled trial (ProtecT Trial) with median 10-year follow up reported prostate-cancer-specific-mortality was low among different treatment groups (AS, Surgery and Radiotherapy) and no significant difference existed in overall survival among the three treatment strategies. “Low risk” prostate cancer was defined as clinical stage T1-T2a (physical examination and imaging), Gleason Score ≤ 6 (biopsy), and prostate specific antigen < 10 ng/mL (blood test) (14). To date, the most comprehensive study of AS in papillary thyroid microcarcinoma (PTMC) was conducted by the Kuma hospital in Japan. In their prospective trial, 8% of 1,235 PTMC patients demonstrated tumor enlargement ≥3 mm and 3.8% demonstrated novel lymph node metastases (LNM) at 10-year follow-up (15). While prognosis for both the immediate surgery and AS cohorts remained excellent, there were significantly less unfavorable events (mainly surgery complications) and medical cost in AS group patients (15). Thus, an increasing number of low-risk PTMC patients in Kuma hospital chose AS as their initial management strategy (16). Per the Kuma hospital criteria, “low risk” PTMC was defined as: no N1 and M1; no sign or symptom of invasion to the recurrent laryngeal nerve (RLN) or trachea; no high-grade malignancy in cytology. In contrast to prostate cancer, this criteria for determining AS candidacy in PTMC was heavily dependent on accuracy of imaging, especially ultrasound. Whether imaging examination could rule out small group of aggressive PTMC from AS candidates reliably remains unknown.

On the basis of these limited data, Leboulleux et al. recommended AS with curative intent should be considered in properly selected PTMC patients (17). However, this suggestion was contested by doctors from United Kingdom, United States, China, and Italy, which meant AS was not equally accepted by all physicians around the world. Clinicians showed little acceptance of AS because they believed evidence to support AS in PTMC was insufficient (18). In contrast to prostate cancer, thyroid cancer patients have better prognoses and lower mortality. However, the utility of AS in thyroid cancer remains controversial. Patients and clinicians alike worry delaying immediate treatment, as would be indicated by AS, may result in more extensive surgical intervention should substantial disease progression occur from the time of initial diagnosis. To address these concerns, it is essential to critically evaluate the ability of diagnostic imaging and biomarkers to accurately stratify risk in PTMC patients.

## Diagnostic Accuracy of Preoperative US

### Extrathyroidal Extension (ETE)

ETE, defined as tumor spread outside of the thyroid gland and into the surrounding tissues, occurs in up to 30% of differentiated thyroid cancer cases (19–23). Minimal ETE, detectable only on histological examination, was not a risk factor for disease specific survival and disease related mortality. Gross ETE, or macroscopic ETE, predicted increased recurrence and mortality (24). Thus, the general consensus is to consider gross ETE as an absolute indication for total thyroidectomy and administration of postoperative radioactive iodine (25). Differentiating minimal from gross ETE is essential in the selection of candidates for AS, however, to date, there is no reliable data to evaluate the diagnostic accuracy of ultrasound (US) alone for gross ETE in PTMC. As shown in Table 1, several studies assessed diagnostic ability of US for ETE (minimal and gross) in PTC or PTMC (26–33). The sensitivity and specificity of US ranged from 25 to 100% and from 13 to 93%, respectively. The huge variation in accuracy of US among different studies may result from : different percentage of minimal and gross ETE; : different diagnostic criteria of US; and : different levels of experience of the US technicians. Furthermore, we extracted 9 cases of T4 PTC patients from 5 articles and found that only 1 patient was diagnosed correctly by US, as shown in Table 2 (30–37). That indicates US alone, which is dependent on the experience of the technician and interpreting physician, can't be used to reliably diagnose gross ETE in PTMC.

**Table 1:** Diagnostic accuracy of preoperative ultrasound for extrathyroidal extension in thyroid cancer.

**References**	**Country**	**Study**	**Patients**	**Criteria**	**SE (%)**	**SP (%)**	**PPV (%)**	**NPV (%)**	**AC (%)**
Shimamoto et al. (26)	Japan	SR	35 of 77 with ETE (minimal and gross)	A	80	73.8	71.8	81.6	76.6
Tomoda et al. (27)	Japan	SR	13 of 509 with TI	C	91	93	25	99	93
Kwak et al. (28)	South Korea	SR	89 of 221 with ETE (N/A)	A	65.2	81.8	70.7	77.7	N/A
Kim et al. (29)	South Korea	SR	67 of 75 with ETE (minimal and gross)	A,C,D	78.5	79.5	46.8	94.1	79.3
Lee et al. (30)	South Korea	SR	174 of 377 with ETE (N/A)	A	66.1	65.1	72.2	58.3	N/A
Lee et al. (31)	South Korea	SR	275 of 568 with ETE (minimal and gross)	A	83.3	68.9	71.6	81.5	75.9
Moon et al. (32)	South Korea	SR	26 of 105 with EFI	E	46.2	97.5	85.7	84.6	84.8
Kamaya et al. (33)	USA	SR	16 of 62 with ETE (minimal and gross)	A,B	25	93	57	78	N/A

*Criteria category: A: focal bulging out or disruption of the thyroid capsule by tumor or more than 25% of perimeter of the tumor was abutting the thyroid capsule; B: vessels extending to or from the nodule were seen beyond the capsule on either color or power Doppler images; C: the absence of a clear adventitia, dilatation of the cartilage space or tumor extension into the space, or irregularity of the tracheal mucosa; D: loss of normal esophageal layer by tumor, the tumor was in contact with 180° or more of the circumference of the vessel and tumor invasion into the vessels lumen or a tumor occupying the tracheal esophageal groove; E: the loss of echo-genic perithyroidal fat tissue by tumor. SR, single center retrospective; ETE, extrathyroidal extension; TI, trachea invasion; EFI, extrathyroidal fat invasion; SE, sensitivity; SP, specificity; PPV, positive predictive value; NPV, negative predictive value; AS, accuracy; N/A, not available*.

**Table 2:** Diagnostic accuracy of preoperative ultrasound for pathologic T4 papillary thyroid carcinoma.

**References**	**Country**	**Study**	**T4 Patients**	**Criteria**	**US accuracy**
King et al. (34)	Hong Kong	SP	3/14 of PTC	A	0/3
Choi et al. (35)	South Korea	SR	1/299 of PTC	B	0/1
Park et al. (36)	South Korea	SP	1/94 of PTC	B,C	0/1
Choi et al. (37)	South Korea	SR	1/722 of PTC	B	1/1
Lee et al. (30)	South Korea	SR	3/568 of PTC	B	0/3

*Criteria category: A: poorly defined margin with heterogeneous echogenicity in adjacent fat or muscle or tumor invasion into the lumen; B: focal bulging out or disruption of the thyroid capsule by tumor or more than 25% of perimeter of the tumor was abutting the thyroid capsule; C, tumor diameter. SP, single center prospective; SR, single center retrospective; PTC, papillary thyroid carcinoma; US, ultrasound*.

Tracheal and RLN invasiveness are the most commonly observed gross ETE. Consequently, the Kuma hospital elected to implement “no signs or symptoms of invasion to RLN or trachea” as their selection criteria for AS in PTMC (15). In 2005, a study from Kuma hospital demonstrated US could diagnose tracheal invasion of PTC with extremely favorable sensitivity, specificity, and accuracy of 91, 93, and 93%, respectively (27). Moreover, Ito from Kuma hospital diagnosed tracheal invasion from low-risk PTMC based on the angles between tumor and tracheal wall with 100% sensitivity and 94.5% specificity, while diagnosis of RLN invasion was based on whether the normal rim of the thyroid was clearly present in the direction of RNL with 100% sensitivity and 90.3% specificity. However, 841 (74%) low-risk PTMC patients in this study were diagnosed with help of plain neck computed tomography (CT) because of uncertainty in US imaging. A study enrolled 377 PTC patients demonstrated the combination of US and CT scan decreased the false negative and false positive rates, improving ETE prediction accuracy. In a subgroup of PTMC, the combination of US and CT features also increased positive predictive value (PPV) remarkably (31). Choi et al. demonstrated that contrast-enhanced CT imaging correctly diagnosed a PTC patient as T4, while US alone would have categorized the patient as T3. However, they indicated the combined use of contrast-enhanced CT imaging and US did not improve accuracy for the diagnosis of minimal ETE in PTMC patients (35).

Currently, there are very few studies reporting RLN invasion in PTMC, presumably due to the low incidence of RLN invasion in PTMC. Ito et al. found only 9 of 1,143 PTMC patients with RLN invasion, all 9 of whom had a tumor diameter of 7 mm or larger. Consequently, Ito et al. concluded tumors of < 7 mm in their largest diameter were unlikely to have RLN invasion. But PTMC was derived from abnormal follicular epithelial cell which meant it could be located anywhere within the thyroid. Small PTMCs (< 5 mm) which invade RLN were more likely located in the dorsal part of thyroid. Inaccurate identification for boundaries of small PTMCs and dorsal membrane of thyroid by US may lead to misdiagnosis of gross ETE. Due to limitations in US at the time of evaluation, ETE in these patients was incorrectly assessed. The evidence reminds us of the limited efficacy for US alone in predicting gross ETE and that not all PTMC patients are low risk.

### Lymph Node Metastases

LNM to the central and lateral compartments are common occurrences in PTC, and increase the rate of loco-regional recurrence and mortality, especially among old patients (38). Nearly 80% of PTC patients had micrometastatic lymph nodes on postoperative pathologic examination while 30% had clinical lymph nodes on initial presentation (39, 40). However, as shown in Table 3, the accuracy of preoperative US for diagnosing metastatic lymph nodes is low (26, 35, 36, 41–52). Appropriate selection of candidates for AS requires high sensitivity in order to prevent the enrollment of higher-risk PTMC patients. To predict central lymph node metastases (CLNM), sensitivity of US ranged from 22.6 to 55%, meaning nearly half of CLNM were not correctly diagnosed. This is perhaps due to the difficulty in detecting metastatic lymph nodes in the retropharynx, superior mediastinum, and deep trachea-esophageal groove. In contrast to CLNM, US sensitivity to detect lateral lymph node metastases (LLNM) was much better (62 to 100%). Of note, micrometastases are less important clinically compared to macrometastases. The benefit of treating incidentally identified micro-metastases are not well-demonstrated. Consequently, the American Thyroid Association (ATA) recommended fine needle aspiration (FNA) only for suspicious cervical lymph nodes larger than 8–10 mm (25). Among the articles we summarized in Tables 3, 5 studies focused on metastatic lymph nodes larger than 8–10 mm (26, 35, 47, 50, 52). However, the sensitivity of US for diagnosing CLNM remained low (26–53.2%). US didn't show any advantages in diagnostic ability for larger metastatic lymph nodes compared with the micrometastases.

**Table 3:** Diagnostic accuracy of preoperative ultrasound for metastatic lymph nodes in thyroid cancer.

**References**	**Country**	**Study**	**Patients**	**Criteria**	**Compartment**	**SE (%)**	**SP (%)**	**PPV (%)**	**NPV (%)**	**AC (%)**
Shimamoto et al. (26)	Japan	SR	49 N1 of 77 PTC	A,B	CLNM, LLNM	36.7	89.3	85.7	44.6	55.8
Jeong et al. (35)	South Korea	SP	46 positive LNs of 312 LNs	A	CLNM, LLNM	53.6	97.9	73.7	95	N/A
Kim et al. (41)	South Korea	SR	53 N1 of 165 PTC	A	CLNM	38	93	77	70	71
					LLNM	64	92	83	82	82
Sugitani et al. (42)	Japan	SP	263 N1 of 361 PTC	A	CLNM	29	91	82	47.3	48.3
					LLNM	100	0	98	0	98
Ahn et al. (36)	South Korea	SR	117 positive levels of 183 cervical level	A	CLNM	55	69	77	44	60
					LLNM	62	79	84	55	68
Choi et al. (43)	South Korea	SR	119 N1 of 299 PTC	A,B	CLNM	53.2	79.8	60.8	74.3	69.9
					LLNM	93.9	25	93.9	25	88.7
Park et al. (44)	South Korea	SR	34 N1 of 94 PTC	A	CLNM	22.6	98.6	87.5	74.5	70.1
					LLNM	76.2	75	72.7	78.3	75.6
Choi et al. (45)	South Korea	SR	238 N1 of 589 PTC	A	CLNM	47.2	94.8	90.4	63.5	70.6
					LLNM	69.1	94.8	57.6	96.8	92.4
Lee et al. (46)	Japan	SR	254 positive LNs of 331 LNs	A	CLNM, LLNM	78	99	99.5	58	83
Hwang et al. (47)	USA	SR	30 N1 of 68 PTC	A,B	CLNM	30	86.8	64.3	61.1	N/A
					LLNM	93.8	80	76.5	94.1	N/A
Lee et al. (48)	South Korea	SR	121 N1 of 252 PTC	A	CLNM	23	97	81	72	73
					LLNM	70	84	81	74	77
Yoo et al. (49)	South Korea	SR	51 positive LNs of 124 LNs	A	CLNM	76.4	69.9	63.9	81	72.6
Lesnik et al. (50)	USA	SP	162 PTC	A,B	CLNM	26	95	78	66	N/A
					LLNM	79	87	80	86	N/A
Lee et al. (51)	South Korea	SR	136 N1 of 368 PTC44 N1 of 48 PTC	A	LLNM	39	88.4	66.3	71.2	70.1
					LLNM	95.5	25	93.3	33.3	89.6
Khokhar et al. (52)	USA	SR	104 N1 of 227 PTC	A,B	CLNM	37.5	90.2	76.5	63.1	66.1

*Criteria category: A: heterogeneous inner structure, loss of fatty hilum, rounded shape, taller-than-wide shape, cystic changes, microcalcifications, and peripheral vascularity; B: Lymph node size >6 mm, or 8 mm, or 1 cm; SR, single center retrospective; SP, single prospective; LN, lymph node; PTC, papillary thyroid carcinoma; CLNM, central lymph node metastasis; LLNM, lateral lymph node metastasis; SE, sensitivity; SP, specificity; PPV, positive predictive value; NPV, negative predictive value; AS: accuracy; N/A, not available*.

**Figure 1:**
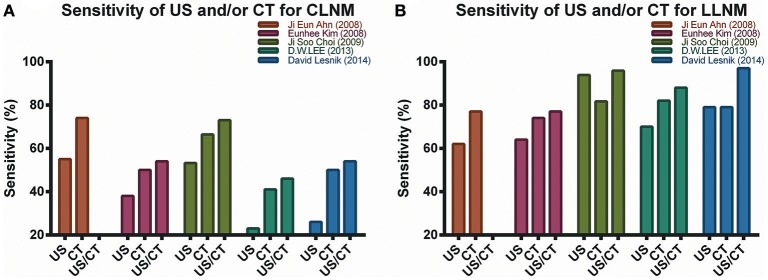
Diagnostic sensitivity was improved by CT alone or combination of US and CT for CLNM **(A)** and LLNM **(B)**. Overall sensitivity of US and/or CT for LLNM was higher than for CLNM. Among Choi (43) and Lesnik (50) studies which only evaluated cervical lymph nodes larger than 10 mm, the combination of US and CT also provided highest sensitivity. The sensitivity for diagnosis of CLNM and LLNM by combination of US and CT was not evaluated in Ahn study (35).

Shown in Figure 1 and Table 4, standalone CT imaging, or CT combined with US remarkably increased CLNM and LLNM diagnostic sensitivity and accuracy (35, 42, 43, 48, 50). In Choi's study which focused on metastatic lymph nodes larger than 10 mm, combination of US and CT increased sensitivity of CLNM from 53.2 to 73%, and LLNM from 93.9 to 95.9% (35). A separate prospective study from United States demonstrated that the combination of US and CT increased sensitivity of detecting metastatic central and lateral lymph node significantly to 54, 97%, respectively. Accordingly, they suggested combination of US and CT could provide reliable preoperative macroscopic nodal metastasis information to design rational nodal surgery in PTC patients (50).

**Table 4:** Diagnostic accuracy of preoperative ultrasound and computed tomography for metastatic lymph nodes in thyroid cancer.

**Study**	**Criteria**	**Compartment**	**SE (%)**	**SP (%)**	**PPV (%)**	**NPV (%)**	**AC (%)**
**Author (year)**: Kim et al. (42) **Country:** South Korea **Type:** SR **Patient:** 53 N1 of 165 PTC	US: A	CLNM	38	93	77	70	71
		LLNM	64	92	83	82	82
	CT:C	CLNM	50	91	79	74	75
		LLNM	74	95	89	86	87
	US+CT: A,C	CLNM	54	84	68	74	72
		LLNM	77	91	84	87	86
**Author (year):** Ahn et al. (35) **Country:** South Korea **Type:** SR **Patient:** 117 of 183 cervical levels	US: A	CLNM	55	69	77	44	60
		LLNM	62	79	84	55	68
	CT: C	CLNM	74	44	72	47	64
		LLNM	77	70	81	64	74
**Author (year):** Choi et al. (43) **Country:** South Korea **Type:** SR **Patient:** 119 N1 of 299 PTC	US: A,B	CLNM	53.2	79.8	60.8	74.3	69.9
		LLNM	93.9	25	93.9	25	88.7
	CT: C	CLNM	66.7	79.3	65.5	80.1	74.6
		LLNM	81.7	100	100	30.8	83.1
	US+CT: A,B,C	CLNM	73	70.2	59.1	81.5	71.2
		LLNM	95.9	25	94	33.3	90.6
**Author (year):** Lee et al. (48) **Country:** South Korea **Type:** SR **Patient:** 121 N1 of 252 PTC	US: A	CLNM	23	97	81	72	73
		LLNM	70	84	81	74	77
	CT: B	CLNM	41	90	66	76	74
		LLNM	82	64	69	78	73
	US+CT: A,B	CLNM	46	88	65	77	74
		LLNM	88	61	69	83	74
**Author (year):** Lesnik et al. (50) **Country:** USA **Type:** SP **Patient:** 162 PTC	US: A,B	CLNM	26	95	78	66	N/A
		LLNM	79	87	80	86	N/A
	CT: C,D	CLNM	50	94	85	74	N/A
		LLNM	79	83	76	86	N/A
	US+CT: A,B,C,D	CLNM	54	89	77	75	N/A
		LLNM	97	77	74	98	N/A

*US criteria category: A: heterogeneous inner structure, loss of fatty hilum, rounded shape, taller-than-wide shape, cystic changes, microcalcifications, and peripheral vascularity; B: Lymph node size < 1 cm; CT criteria category: C: round shape, calcification, cystic or necrotic change, heterogeneous enhancement, and strong enhancement without hilar vessel enhancement; D: Short axis >1 cm in axial plane; SR, single center retrospective; SP, single prospective; PTC, papillary thyroid carcinoma; US, ultrasound; CT, computed tomography; CLNM, central lymph node metastasis; LLNM, lateral lymph node metastasis; SE, sensitivity; SP, specificity; PPV, positive predictive value; NPV, negative predictive value; AS, accuracy; N/A, not available*.

The limitations of US to detect thyroid nodule and cervical lymph nodes were operator-dependent, presumably due to difficultly in evaluating deep anatomic structures such as mediastinum, parapharyngeal, retropharyngeal and infraclavicular regions, acoustically shadowed by bone, calcification or air (66). As a result, the 2015 ATA guideline recommended preoperative CT as an adjunct to US for patients with large or invasive primary tumor or US suspected advanced disease (25). Nevertheless, is it possible to diagnose gross ETE with virtually 100% sensitivity, as reported by the Kuma hospital? Could we diagnose cervical macroscopic LNM with decent sensitivity only by US? As summarized above, it may be possible to approach this high sensitivity through the combination of diagnostic CT and US imaging, which demonstrated significant improvements in diagnostic performance compared to US alone.

### Radiologist vs. Surgeon Performed US

Multiple studies demonstrated that radiologist-performed USs were less accurate and provided inadequate preoperative staging when compared to surgeon-performed USs (67–70). Nearly half of patients received incorrect initial surgery with high local recurrence when an operation decision was made only based on radiologist-performed USs. Denise Carneiro-Pla reported surgeon-performed US changed the therapeutic strategy of 45% thyroid cancer patients through the accurate identification of CLNM/LLNM and thyroid intrathoracic extension (69). Rosebel Monteiro demonstrated that metastatic lymph nodes were diagnosed more frequently by CT imaging than US (70.8 vs. 54%). Moreover, surgeon-performed US was only able to detect 45% of metastatic lymph nodes in a cohort comprised of patients with LLNM (67). In the Kuma hospital, US was performed by specially trained sonographers and retrospectively reviewed by surgeons (15). Thus, it is extremely important to note that the appropriate selection of low-risk PTMC patients for AS is limited by the experience, or inexperience, of diagnosing physicians. Addressing this issue means improvements in both imaging technologies and in the education of physicians play important roles in AS candidate selection.

## Novel LNM During AS

Sixteen percent of AS patients will require surgical intervention due to disease progression (71, 72). However, despite disease progression, prognosis in these patients remains remarkably excellent due to the success of salvage surgery (15). In certain circumstances, most commonly the appearance of a novel LNM in the lateral/central compartment, delayed surgical intervention may increase the risk of subjecting a patient to more invasive surgical procedures, an increased risk of recurrence, and more extensive follow-up. A retrospective study which enrolled 8,808 PTMC in Korea demonstrated 12 PTMC with distant metastases had cervical lymph node involvement. Among them, 10 patients had clinically apparent lateral lymph nodes, while 2 had microscopic CLNM (73). Xu et al. investigated 3,750 non-anaplastic follicular cell-derived thyroid carcinomas and found that, of the 3 PTMC-related deaths, all 3 patients had clinically apparent cervical lymph nodes (74). Clinically apparent cervical lymph nodes were positively related with recurrence, distant metastases, and disease-specific mortality. Consequently, patients in which cervical lymph node involvement is detected during follow-up, no longer benefit from AS, and additional therapeutic intervention should be explored. According to a series of studies from Kuma hospital, the rates of novel lymph node appearance among patients undergoing AS were 1.2, 1.5, and 2.1% with an average of 3.9, 5, and 6.2 years of observation, respectively (72, 75, 76). Ito et al. explained that LNM may occur prior to, or at a very early stage of, PTMC diagnosis. Consequently, immediate surgery will not prevent metastases to the neck lymph node(s) and these patients will have recurrence and require a salvage operation in the future regardless of the initial management strategy (15). However, it is debated as to whether novel LNM during AS are completely comparable to recurrent lymph node involvement in an immediate surgery cohort, as it is difficult to demonstrate whether the tumor cells disseminate into lymph node during AS or before diagnosis.

If early dissemination of tumor cells to a regional or distant lymph node has occurred prior to diagnosis and/or initial thyroidectomy, excision of the primary thyroid cancer is unlikely to prevent recurrent disease localized to the lymph nodes. The parallel progression hypothesis, defined as the capacity of tumor cells to spread to the lymph nodes or more distant sites from the primary tumor site at a very early stage of tumorigenesis leading to the independent progression/evolution of a metastatic site, may explain early dissemination and frequent lymph node recurrence after surgery (77). PTC recurs much more frequently at central or lateral lymph node than thyroid bed after surgery (55, 78, 79), suggesting that recurrent lymph nodes of the neck had an early dissemination event prior to excision of the primary PTC.

Microscopic metastasis in regional lymph nodes was present in up to 63.83% of PTMC patients, although the recurrence rate was much lower to 1–5% (17, 80). The mechanism of lymph node recurrence after initial surgery without prophylactic lymph node dissection was possibly the outgrowth of micro-metastatic deposits into overt tumors. Whether or not a population of microscopic tumor cells can transform into clinically apparent lymph nodes may depend on not only the intrinsic genetic alterations of the cancer cells themselves but also the state of the host environment (81). It is well-known that both the local tissue microenvironment and the systemic physiological environment play significant roles in regulating dormant disseminated tumor cells into gross metastasis. Additionally, the tumor microenvironment can change during multiple steps of tumor progression and metastases, which could either inhibit or facilitate the progression of microscopic lymph nodes to clinically apparent lymph nodes (82–84). Perhaps, however, there exists a connection between persistent tumor foci in the thyroid and the progression of LNM from microscopic to clinically apparent.

**Figure 2:**
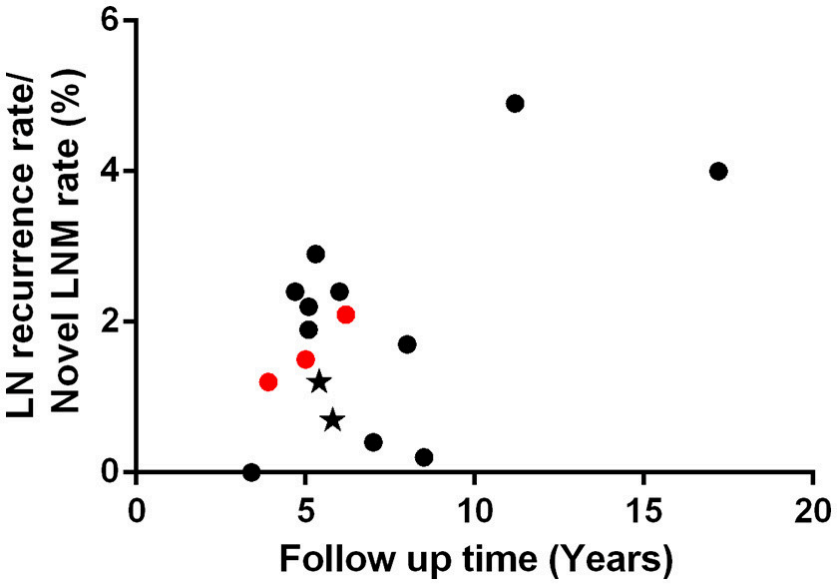
Cervical lymph node recurrence rates (black dots and stars) among 13 different PTMC cohorts and novel LNM rates (red dots) in AS groups. With < 10-year follow up, 5 PTMC cohorts had relatively higher lymph node recurrence than novel LNM rate in AS patients because all of these 5 cohorts had small group of PTMC with gross ETE and/or clinical apparent lymph node (detail seen Table 5). The lymph node recurrence rates of “low-risk” cohorts (black stars), which excluded patients with gross ETE and clinical apparent lymph node, were relatively less than novel LNM rates in AS groups.

We summarized 13 PTMC cohorts, each containing more than 200 patients, who received immediate surgery, shown in Table 5 (53–65). We hypothesize, that AS patients should have a lower rate of novel clinically apparent metastatic lymph nodes than the rate of recurrent lymph nodes in PTMC cohort with gross ETE and/or palpable lymph nodes. However, as shown in Figure 2, we found novel LNM during AS was not less than lymph node recurrence rate among 6 of 11 PTMC cohorts with median follow-up time <10 years. Meanwhile, 5 of these 11 cohorts had relatively higher lymph node recurrence rates than AS group because all of these cohorts had patients with gross ETE and clinical involved lymph nodes. Among these 13 PTMC cohorts, there were two studies which enrolled low-risk PTMC patients without gross ETE or clinically apparent lymph nodes. Their lymph node recurrence rates were 1.2 and 0.7% with 5.4- and 5.8-year follow up, which were less than 1.5 and 2.1% of novel lymph node appearance rate with 5- and 6.2-year observation time in AS cohort from Kuma hospital (64, 65, 72, 76). With limited data, the rate of clinical apparent LNM in low-risk PTMC patients under AS seems to be a little higher than patients with immediate surgery. Considering cofounders between different patient's cohorts, this preliminary result needs to be supported and proved by more evidence in the future. Oda et al. compared clinicopathological and prognostic features of low-risk PTMC between AS and immediate surgery groups with a comparable experimental timeline. They found novel LNM appeared in 6 of 1179 AS patients (0.5%), whereas only 2 of 974 (0.2%) patients choosing immediate surgery experienced recurrence in cervical lymph nodes although this difference was not statistically significant (85). A study from Italy which enrolled 312 very low-risk PTMC (No family history of thyroid cancer; No history of head and neck irradiation; Tumor staging: T1 1 cm or less, N0, M0; No extension beyond thyroid capsule; Unifocal; Not aggressive histologic subtype; Not locally invasive) with 6.7-year follow up demonstrated none of the patients had lymph node recurrence (86). In addition, another study from Kuma hospital found up to 11% of PTMC in cohort of young patients aged 20 to 29 had novel LNM with median 5.5-year follow up (87). If novel LNM in AS group was completely comparable with lymph node recurrence in an immediate surgery cohort, should 11% of low-risk PTMC in 20 s group who underwent immediate surgery have lymph node recurrence after 5.5-year follow up? Patient age was believed to be predictor for novel lymph node appearance during AS (72, 87). However, age was not a risk factor for cervical lymph node recurrence in PTMC patients (79, 88).

**Table 5:** Cervical lymph node recurrence rate in different papillary thyroid microcarcinoma cohorts with immediate surgery.

**References**	**Country**	**No. of patients**	**Gross ETE (n, %)**	**Clinical LN (n, %)**	**RAI (n, %)**	**FU (years)**	**TR (n, %)**	**LNR (n, %)**
Wada et al. (53)	Japan	259	N/A	24 (9.3)	N/A	5.1	6 (2.3)	5(1.9)
Pelizzo et al. (54)	Italy	403	N/A	N/A	260 (60.5)	8.5	6(1.5)	1(0.2)
Hay et al. (55)	USA	900	N/A	131 (14.6)	155 (17)	17.2	51(5.7)	36 (4)
Besic et al. (56)	Slovenia	254	N/A	51 (20.1)	124 (49)	4.7	7 (2.7)	6(2.4)
Mercante et al. (57)	Italy	445	N/A	37 (8.3)	389 (87.4)	5.3	17(3.8)	13(2.9)
So et al. (58)	South Korea	551	4 (0.7)	0	444 (80.6)	3.4	1(0.2)	0
Moon et al. (59)	South Korea	288	0	10 (3.5)	114 (39.6)	6	12 (4.2)	7(2.4)
Londero et al. (60)	Denmark	406	N/A	N/A	161(40)	8	15(3.7)	7(1.7)
Lee et al. (61)	South Korea	2014	18 (0.9)	N/A	51(2.5)	11.2	126(6.3)	98(4.9)
Gschwandtner et al. (62)	Austria	1391	N/A	N/A	255 (18.3)	7	5(0.4)	5(0.4)
Kim et al. (63)	South Korea	5656	210 (3.7)	N/A	N/A	5.1	126(2.2)	122(2.2)
Cecoli et al. (64)	Italy	437	0	0	152 (38.7)	5.8	6(1.4)	3(0.7)
Kim et al. (65)	South Korea	8676	0	0	3,863 (44.5)	5.4	139(1.6)	105 (1.2)

*ETE, extrathyroidal extension; LN, lymph node; RAI, radioactive iodine; FU, follow up; TR, total recurrence; LNR, lymph node recurrence; N/A, not available*.

In contrast to AS, the benefits of immediate surgery may include: ① A more accurate risk stratification can be made using information gathered from histological or genetic evaluation of a biopsy obtained from surgery, than can be obtained from imaging data alone. ② TSH suppression after surgery would decrease recurrence risk in contralateral lobe and neck lymph node. ③ Serum Tg is an accurate and reliable biomarker of tumor burden in Tg auto-antibody negative patients who received a total thyroidectomy. ④ For PTMC patients with lymph node recurrence, metastatic lymph nodes were stable for many years (89). At this time, it may be more feasible to use serum Tg levels during AS for monitoring recurrent lymph nodes.

## Ethical Issues

In 2000, Emanuel et al. argued the most important ethical concerns in clinical trials was “the potential benefits to individuals must outweigh the risks (90).” However, with only US and FNA, we have little prognostic information, with the exception of age and tumor size, to evaluate the safety of AS. Consequently, Stack and Angelos recommended implementing only institutional review board-approved research protocols or surveillance contracts for educating patients, codifying the relationship between clinician and patient, and establishing medicolegal protections (91). But Morris et al. disagreed, instead believing these documents would jeopardize patient autonomy and influence their choice (92). Supporters of AS think higher risk among a small number of patients will and should be balanced by the advantage of avoiding surgery in a larger number of patients (93). However, is it ethical to risk the health of some patients, even a minority, for the greater good? Recently, Dr. Akira estimated the lifetime disease progression probabilities, stratified by patient age, of PTMC during AS, which were 60.3% (20 s), 37.1% (30 s), 27.3% (40 s), 14.9% (50 s), 9.9% (60 s), and 3.5% (70 s) (87). This study provided significant information for AS patients selection. In the future, we need more information from imaging and molecular signatures to provide more accurate risk stratifications of the clinical behavior and the risk for disease progression of PTMC patients during AS.

## Improvements in Imaging

### US

In terms of diagnostic accuracy, 3-dimensional (3D) US outperformed 2-dimensional (2D) US when compared to patients' final histopathological outcome (94). A single sweep of 3D US provided imaging for reconstruction and overcame the major limitations of 2D US. Kim et al. evaluated 91 thyroid nodules from 85 consecutive patients and compared sensitivity and specificity between 3D and 2D US. They found 3D US had significantly higher sensitivities than 2D in predicting ETE (94). In contrast, a separate study from South Korea reported 3D US with tomographic ultrasound imaging algorithms alone was not superior to real-time 2D US (95). This discrepancy is perhaps attributable to the differences that variable image reconstruction parameters have on US interpretation. Slapa et al. summarized the advantages of 3D US as follows: distinct separation between imaging acquisition and analysis, better remote consultation, less operator dependency, and increased diagnostic accuracy (96).

Recently, shear wave elastography (SWE) has emerged to diagnose and predict the pathologic prognostic factors of PTC using quantitative information about thyroid nodule elasticity. It is operator-independent and can display elastograms of estimated tissue stiffness. Yun et al. enrolled 208 PTC patients and found ETE was associated with the elasticity index determined by SWE, and quantification of the elasticity index could accurately predict pathologic ETE (97). Diagnostic accuracy of cervical lymph nodes was also significantly improved by SWE. Woo et al. reported the elasticity indices of SWE were significantly correlated with not only malignant lymph nodes, but also the number, size and ETE of involved lymph nodes. They concluded quantitative SWE could predict pathologic prognostic factors of cervical LNM (98). Azizi et al. evaluated 270 lymph nodes from 236 patients with both conventional US and SWE. Using single shear wave velocity cut off of 2.93 m/s, SWE could improve diagnostic sensitivity and specificity to 92.59 and 75.46%, respectively. Lymph node stiffness measured by SWE is reported to be an independent predictor of malignant lymph node (99). Xu from China also found predictive performance for CLNM in PTC was markedly improved with the combination of conventional US and SWE, which indicated SWE would be a useful tool for treatment planning (100).

### CT and MRI

Liu et al. evaluated cervical metastatic lymph nodes using dual-energy spectral CT and found venous phase λ_HU_ (slope of the spectral Hounsfield unit curve) was the best parameter for diagnosis with sensitivity, specificity of 62.0 and 91.1%, respectively. Compared to conventional CT, quantitative assessment with gemstone spectral CT parameters improved accuracy for detecting cervical metastatic lymph nodes of PTC (101). Considering MRI, several studies have reported the apparent diffusion coefficient (ADC) derived from diffusion-weighted imaging (DWI) could be used as a predictor for thyroid cancer aggressiveness (102–104). Hao et al. evaluated the predictive performance of ADC for ETE of PTCs in a cohort of 23 PTMC patients. PTCs with ETE had significant lower median ADC, 5th percentile ADC, and 25th percentile ADC while PTMCs had significant lower ADC only in 5th percentile ADC (102). Another study used DWI histogram analysis of whole tumor ADC to investigate the relationships between ADC parameters with histopathological features like LNM, ETE, Ki-67, and p53. They found ADC mean, ADC max, ADC median, ADC modus, ADC p75, and ADC p90 were all related significantly with p53, which was prognostic marker for thyroid cancer. Moreover, they identified an inverse correlation between ADC max, ADC p90, and Ki-67, which was regarded as predictor for disease progression during AS (105). Importantly, ADC histogram skewness and kurtosis were also identified to be parameters for predicting LNM (104). Meyer et al. demonstrated MRI texture analysis, which was a novel imaging technique derived from extensive data provided by conventional sequences, was a very useful tool to predict histopathological features in thyroid cancer although they only enrolled 13 thyroid cancer patients (4 PTC; 4FTC; and 5 ATC) (103).

## Improvements in Biomarker

### BRAF

Braf, as a member of RAF kinase family, served as a growth signal transduction protein kinase. Braf^V600E^ composed nearly 90% of all somatic mutated Braf and played an important oncogenic role in thyroid tumorigenesis (106). The replacement of valine with glutamate at codon 600 resulted from the substitution of thymine with adenine at nucleotide 1799, then activated its serine/threonine kinase constitutively, leading to further activation of MAPK pathway (107). The downstream effectors of mutated Braf, such as Mek and Erk, will be phosphorylated and take part in thyroid tumorigenesis (106, 107). Moreover, Braf^V600E^ could promote tumor formation and aggressiveness by regulating the expression of other genes epigenetically, either through hyper- or hypomethylation. The interaction between Braf^V600E^ and epigenetic alterations, which downregulated tumor suppressor genes (like T1MP3, SLC5A8, DAPK1, RARβ2) and upregulated oncogenes (like HMGB2 and FDG1), increased tumor cell proliferation and invasion (108, 109).

In 2015, a meta-analysis was performed to investigate the correlation between Braf^V600E^ and clinical features for PTMC (110). In Li et al, the authors analyzed 3437 PTMC patients across 19 studies after searching PubMed, EMBASE, and the Cochrane library. They found that Braf^V600E^ mutation was associated with aggressive clinicopathological features like multifocality, ETE, LNM, and advanced stage of PTMC. Consequently, they suggested Braf^V600E^ could be used as a risk factor for the stratification and management of PTMC (110). Lee et al. predicted gross ETE of PTMC with 100% sensitivity through the use of tumor size, US features, and Braf^V600E^ mutation status. They categorized US features of the primary tumor into four groups: A: intraparechymal; B, tumor abutting the capsule < 50% of diameter; C: tumor abutting >50% of diameter; and D: tumor destroyed the capsule. In a subgroup of Braf^V600E^negative patients, a tumor size of 0.7 cm and US categorizations B and C were cut-off values for gross ETE, with 100% sensitivity, whereas US categorizations A and B as cutoff value had 100% sensitivity for predicting gross ETE in the Braf^V600E^ mutation positive patients (111). Besides clinical risk features, Chen et al. also found PTMCs with Braf^V600E^ mutation were more likely to recur (OR 2.09 [95% CI:1.31–3.33]) by a meta-analysis of 2,247 PTMC patients from 4 published studies and 2 institutional cohort primary data (112). Niemeier et al. developed a molecular-pathological score (including superficial tumor location, intraglandular tumor spread/multifocality, tumor fibrosis, and Braf status) to stratify PTMC into different risk groups and successfully predict recurrence rate. In the diagnosis of aggressive PTMC, the combination of histologic features and Braf status increased diagnostic sensitivity from 77 to 96% and specificity from 68 to 80% (113). With mounting evidence, revisions to the ATA guidelines in 2015 began to consider Braf^v600e^ status as a risk factor of structural disease recurrence in PTMC patients after initial therapy (25).

However, Miyauchi et al. in the Kuma hospital detected BRAF^V600E^ status in 11 PTMC patients without disease progression, 10 PTMC with tumor size progression, and 5 with novel LNM. The percentage of Braf^V600E^ was 64, 70, and 80% in each group, respectively (114). Consequently, the use of BRAF^V600E^ alone is insufficient to accurately stratify risk in PTMC patients. If using Braf^V600E^ alone as biomarker for selecting AS candidates, nearly 60% of PTMCs who may never have disease progression will be categorized wrongly. Considering high prevalence of Braf^v600e^ mutation among PTMC, Braf^V600E^ alone cannot be used as reliable biomarker for differentiating aggressive PTMC from indolent ones, and identifying potential disease progression cases from stable ones during AS. A possible reason may be that the oncogenic event driving PTMC aggressiveness requires additional mutations acting in conjunction with BRAF^V600E^ and the MAPK signaling pathway (115). Thus, the identification of additional genetic variants, which are less abundant than BRAF^V600E^, could be important in predicting PTMC aggressiveness.

### TERT

Telomerase reverse transcriptase (TERT) is the catalytic protein subunit of telomerase, which can maintain chromosomal integrity and genome stability (116). Malignant cancer cells, which were replicative immortal, required activation of telomerase and regulation of other growth controlling genes, pathways and molecular by TERT (117). First reported in 2013, TERT promoter mutation (C228T and C250T) in thyroid cancer has progressed rapidly in recent 5 years (118). Many studies have demonstrated TERT mutation was associated with more aggressive clinicopathological features of thyroid cancer, such as male gender, ETE, LNM, advanced stage, distant metastasis, recurrence, and mortality (119–122). Two meta-analyses in 2016 investigated clinicopathological significance of TERT promoter mutations in PTC and found the average prevalence of TERT promoter mutation was around 10%. Additionally, PTC patients with TERT promoter mutation displayed more aggressive histopathological features (121, 122). Kim et al. developed an effective risk stratification system using TERT promoter mutation status that reliably predicted structural recurrence and mortality in DTC patients (119).

Of note, the co-occurrence of Braf and TERT promoter mutations enhanced the predictive ability for prognosis of PTC. Moon et al. performed a meta-analysis including 13 studies with 4,347 PTC patients and found the co-occurrence of Braf and TERT promoter mutations was more significantly associated with aggressive clinicopathological features than either mutation alone (123). Accordingly, they believed these two mutations had a synergistic effect on prognosis and were useful in risk stratification of PTC. Liu et al. categorized 1,051 PTC patients according to mutation status of Braf and TERT promoter and demonstrated deaths per 1,000-person years in PTC patients with neither mutation, Braf ^V600E^ alone, TERT mutation alone, or both mutations were 0.80, 3.08, 6.62, and 29.86, respectively. Simple 4-genotype classification can predict disease-specific mortality accurately (124). Recently, this synergistic effect of BRAF and TERT promoter has been demonstrated as Braf^V600E^ → MAPK pathway → FOS → GABP → TERT signaling/transcription axis in human cancers (125). Firstly, mutated Braf^V600E^ activated MAPK pathway, which phosphorylated FOS to be an active transcription factor for activating the GABPB promotor. Then increased expression of GABPB and formation of GABPA-GABPB complex activated the mutant TERT promoter. In this axis, phosphorylated FOS played important oncogenic bridging role between Braf^V600E^ and TERT promoter mutations (125).

However, de Biase et al. detected TERT promoter mutations with next-generation sequencing in 431 PTMC patients assembled from six different institutions. They found the prevalence of TERT promoter mutations among PTMC was only 4.7%, less than the 10% reported in PTC patients. Moreover, the presence of TERT promoter mutations was not associated with unfavorable clinicopathological features (126). Also in Miyauchi's study, no PTMC patients undergoing AS were positive for TERT promoter mutations, even in a subgroup of patients with increased tumor sizes and/or novel lymph node appearance (114). Therefore, with regard to its low prevalence in PTMC, TERT promoter mutations are unlikely to be reliable molecular markers of tumor aggressiveness/progression.

### MicroRNA

MicroRNA is defined as a group of small endogenous, single stranded non-coding RNAs of 19–25 nucleotides that can exclusively regulate their proprietary mRNA expression (127). The miRNA-221-222 cluster, downstream of the MAPK pathway, played an important role in tumorigenesis and aggressiveness for PTC (128). Located on the X chromosome, miRNA-221-222 cluster was regulating PTC formation and invasion through negative regulation of p27 (129). Multiple studies have demonstrated that upregulated miR-221-222 cluster was associated with more unfavorable clinicopathological features, treatment resistance, increased recurrence rate, and worse prognosis (130–133). Because of that, the miRNA-221-222 cluster was considered as a potential biomarker for aggressive PTC. Additionally, miRNA-146b is another well-studied and overexpressed microRNA in PTC. Its expression level was positively associated with tumor aggressiveness and poor prognosis (131, 133). Study has shown miRNA-146b functioned in PTC through binding with the 3′UTR region of retinoic acid receptor beta (RARβ) (134). Moreover, advanced PTC patients could receive benefit from retinoic acid (a RARβ ligand) treatment. Retinoic acid treatment resulted in tumor shrinkage and increased radioiodine uptake in 38% and 26% of patients, respectively (135). These studies suggested that miRNA-146b might play important role in thyroid cancer initiation and progression. In addition to the two microRNAs discussed above, there are also other microRNAs which have been identified to be associated with tumor aggressiveness (especially ETE, LNM and distant metastasis) including miRNA135-b, 146-a, 146-5p and several others (Table 6) (129, 136–155).

**Table 6:** Tissue microRNA as predictor for aggressiveness in papillary thyroid carcinoma.

**MicroRNA**	**Change in APTC**	**ETE**	**LNM**	**DM**	**Target molecular**	**References**
MiR-126-3p	↓	*			ADAM9,SLC7A5	(136)
MiR-130b	↓	*			N/D	(137)
MiR-135b	↑	*			N/D	(138)
MiR-146a	↑	*	*		RARβ,PRKCE	(137, 139–142)
MiR-146b	↑	*	*	*	KIT, SMAD4, ZNRF3,IRAK1, RARβ	(137–140, 142–148)
MiR-16	↓	*			ITGA2	(145)
MiR-199b-5p	↑	*	**		N/D	(149)
MiR-221	↑	*	*	*	p27,TIMP3	(129, 132, 137, 138, 143, 145, 150)
MiR-222	↑	*	*		p27, PPP2R2A,TIMP3	(129, 132, 137, 138, 143, 145, 151)
MiR-2861	↑		**		N/D	(152)
MiR-30a-3p	↓		*		N/D	(149)
MiR-34b	↓	*			N/D	(137)
MiR-363-3p	↓		*		PIK3CA	(153)
MiR-451	↑		**		N/D	(152)
MiR-613	↓	*			FN1	(145)
MiR-622	↓		*		VEGFA	(154)

*APTC, aggressive papillary thyroid carcinoma; ETE, extrathyroidal extension; LNM, lymph node metastases; DM, distant metastases; N/D, not determinated*.

↑ *up-regulated in aggressive PTC*.

↓ *down-regulated in aggressive PTC*.

* *related with aggressive features*,

** *Related with central and lateral neck lymph node metastases*.

The upregulation of miRNA-221-222 cluster and miRNA-146b in BRAF^V600E^ positive tumors, was suggested to be attributable to activation via the NF-κB pathway (156, 157). In Braf^V600E^ PTMC patients, it remains unknown what molecular events trigger disease progression during AS. Would it be possible to increase our ability to predict PTMC disease progression by screening FNA biopsies for clinically actionable somatic mutations and/or the expression of miRNAs?

### Serum Circulating Biomarkers

Compared with inherent instability of mRNA, circulating miRNA is subjected to nuclease activity and resistant to environment. Because of that, miRNA, which can be readily detected in bloodstream, is believed as a potential ideal candidate serum biomarker for PTC (158). Yu et al. detected serum miRNA expression by Solexa sequencing and found increased miR-151-5p, detected in the serum, was associated with LNM of PTC (159). However, the evidence of using circulating miRNA to predict disease progression of PTMC during AS was absent. In addition, a prospective observational pilot study found circulating myeloid-derived suppressor cells, which were detected preoperatively by novel flow cytometry-based immunoassay, were positively associated with a higher TNM stage and disease recurrence (160). Lubitz et al. reported they only detected 63% circulating Braf^V600E^ mutation by novel RNA-based blood assay compared with conventional tissue assays on surgical specimens. They concluded detecting circulating Braf^V600E^ could be a surrogate for conventional FNA detection (161). In contrast, a separate study found only 37.3% of PTC patients with locally advanced and metastasis were detected to have circulating Braf^V600E^ mutation. These patients didn't get any benefits from analysis of circulating tumor DNA (162). Accordingly, there are several challenges about the application of serum circulating biomarkers for PTMC which include: ① Molecular FNA diagnostics with biomarkers have high concordance with pathological results. In contrast, serum circulating biomarkers demonstrate only partial concordance with FNA determined pathology. Consequently, circulating biomarkers from blood are not superior to FNA biopsies in predicting aggressiveness. ② All studies about detecting serum circulating biomarkers enrolled cancer patients with advanced stage or distant metastasis. However, the serum circulating biomarkers identified in high-risk patients may not be detectable in low-risk PTMC patients. ③ Genetic background and alternations in circulating cells may be different with those in the primary tumor. Some cancer cells derived from the primary tumor may undergo changes that facilitate blood vessel invasion and then turn to circulating cells. ④ Other malignant tumors shared the same circulating miRNA or DNA with thyroid cancer. Differentiating where these circulating biomarkers came from is difficult.

### Other Novel Targets

Besides genetic alternations, LncRNAs, which is defined as a class of RNAs containing over 200 nucleotides, play important roles in tumor progression (163). Kim et al. reported LOC100507661 expression was positively related with LNM and Braf^V600E^ mutation in PTC patients (164). High expression of HOTAIR in thyroid cancer was associated with larger tumor size, more metastatic lymph nodes, and poorer outcome after a meta-analysis of TCGA and GEO databases (165). PTC patients with high expression of HIT000218960 had more multifocality, LNM and advanced TNM stage (166). Down-regulation of LINC00271 was identified as an independent risk factor for ETE, LNM, TNM stage and recurrence (167). Other LncRNAs, which related with aggressiveness of PTC, were also identified and summarized in Table 7 (164, 166–183).

**Table 7:** Long Non-coding RNA as predictor for aggressiveness in papillary thyroid carcinoma.

**LncRNA**	**Change in APTC**	**ETE**	**LNM**	**DM**	**Target molecular**	**References**
ATB	↑		*		N/D	(167)
CASC2	↓		*		N/D	(168)
CNALPTC1	↑		*		miR-30 family	(169)
GAS8-AS1	↑		*		N/D	(170)
HIT000218960	↑		*		HMGA2	(165)
HOXD-AS1	↑		*	*	N/D	(171)
LINC00271	↓	*	*		N/D	(166)
LINC01061	↑		*		miR-4316	(172)
LOC100507661	↑		*		N/D	(163)
MALAT1	↑		*		N/D	(173)
MEG3	↓		*		Rac1	(174)
NONHSAT037832	↓		*		N/D	(175)
NONHSAT076754	↑		*		N/D	(176)
NONHSAT129183	↑		*		N/D	(177)
NONHSAT076747	↑		*		N/D	(178)
NONHSAT122730	↑		*		N/D	(178)
NR_036575.1	↑	*			N/D	(179)
PVT1	↑	*	*		IGF1R	(180)
RP11-402L6.1	↑		*		N/D	(181)
XLOC_051122	↑		*		N/D	(182)
XLOC_006074	↑		*		N/D	(182)

*APTC, aggressive papillary thyroid carcinoma; ETE, extrathyroidal extension; LNM, lymph node metastases; DM, distant metastases; N/D, not determinated*.

↑ *up-regulated in aggressive PTC*.

↓ *down-regulated in aggressive PTC*.

* *related with aggressive features*.

Epigenetic changes, particularly methylation of DAPK, REC8, TIMP3, CDH1, FGFR2 were also reported to be associated with aggressive behavior of PTC (184). Whether we can predict the aggressiveness of PTMC using these biomarkers derived from PTC patients remains to be investigated.

## Conclusion

The utility of AS for low-risk PTMC patients requires improvements our abilities to accurate and confidently stratify patient risk. Due to the substantially improved diagnostic performance in identifying gross ETE and macroscopic cervical LNM, the combined use of US and CT imaging modalities is strongly recommended for use in AS. Patients should be informed and educated fairly and objectively according to the data that is currently available. Dynamic monitoring, risk stratification, and personal follow-up schedules are tantamount in minimizing the potential risks incurred by recommending patients against immediate surgery. Furthermore, the advent of increasingly sophisticated imaging technologies, and the screening for novel prognostic biomarkers have shown great promise, although future validation studies are warranted.

## Author Contributions

All authors listed have made a substantial, direct and intellectual contribution to the work, and approved it for publication.

### Conflict of interest statement

The authors declare that the research was conducted in the absence of any commercial or financial relationships that could be construed as a potential conflict of interest.
